# Aminolevulinic Acid (ALA) as a Prodrug in Photodynamic Therapy of Cancer

**DOI:** 10.3390/molecules16054140

**Published:** 2011-05-19

**Authors:** Małgorzata Wachowska, Angelika Muchowicz, Małgorzata Firczuk, Magdalena Gabrysiak, Magdalena Winiarska, Małgorzata Wańczyk, Kamil Bojarczuk, Jakub Golab

**Affiliations:** 1 Department of Immunology, Centre of Biostructure Research, Medical University of Warsaw, Banacha 1A F Building, 02-097 Warsaw, Poland; 2 Department III, Institute of Physical Chemistry, Polish Academy of Sciences, 01-224 Warsaw, Poland

**Keywords:** 5-aminolevulinic acid, photodynamic therapy, cancer, laser, singlet oxygen

## Abstract

Aminolevulinic acid (ALA) is an endogenous metabolite normally formed in the mitochondria from succinyl-CoA and glycine. Conjugation of eight ALA molecules yields protoporphyrin IX (PpIX) and finally leads to formation of heme. Conversion of PpIX to its downstream substrates requires the activity of a rate-limiting enzyme ferrochelatase. When ALA is administered externally the abundantly produced PpIX cannot be quickly converted to its final product - heme by ferrochelatase and therefore accumulates within cells. Since PpIX is a potent photosensitizer this metabolic pathway can be exploited in photodynamic therapy (PDT). This is an already approved therapeutic strategy making ALA one of the most successful prodrugs used in cancer treatment.

## 1. Introduction

**Figure 1:**
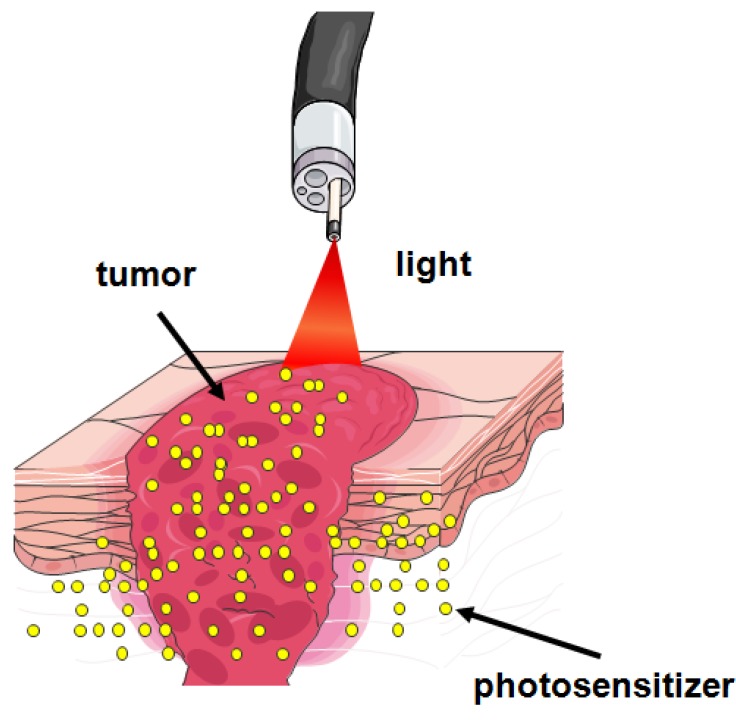
Overview of PDT. Following photosensitizer administration it undergoes systemic distribution and selectively accumulates in the tumor. Illumination activates the photosensitizer and in the presence of molecular oxygen triggers a photochemical reaction that culminates in the production of ^1^O_2_.

Photodynamic therapy (PDT) is a minimally invasive therapeutic modality used in the management of various cancerous and pre-malignant diseases. It involves the systemic administration of a non-toxic photosensitizing (PS) drug, which accumulates in host and tumor cells, and subsequent illumination of the tumor site with visible light, corresponding to the appropriate photosensitizer absorption wavelength (Figure 1). 

**Figure 2:**
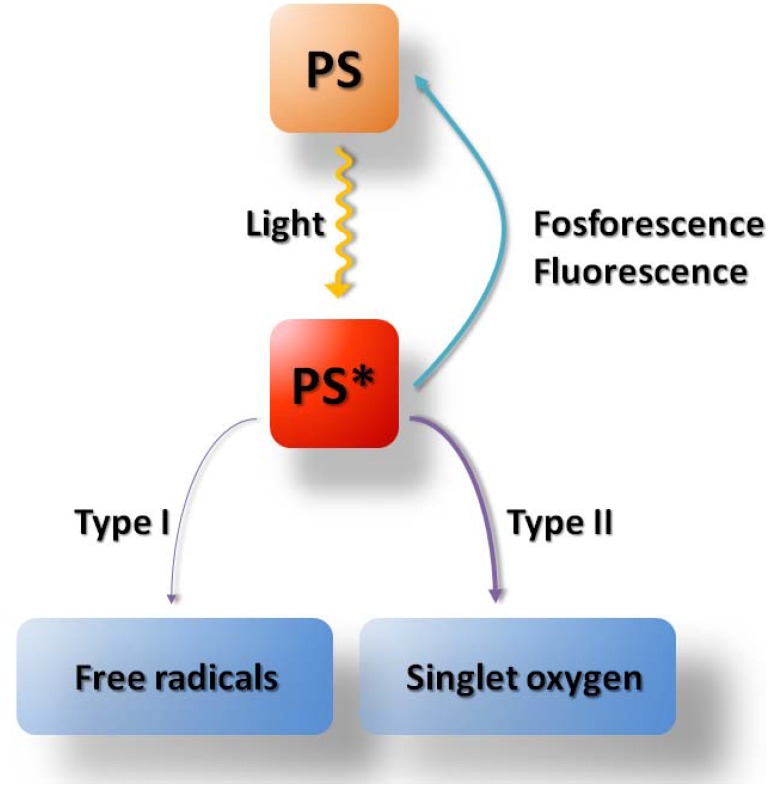
Types of oxidative reactions during PDT. Light with sufficient energy and wavelength matching absorption spectrum of the photosensitizer (PS) can activate a photochemical reaction that leads to formation of activated PS molecule (denoted by asterisk). Activated PS can lose its energy by emitting of visible light (fosforescence or fluorescence). Alternatively it may generate singlet oxygen in a type II reaction or free radicals in a type I reaction.

The excited photosensitizer contributes to the generation of singlet oxygen and other reactive oxygen species (Figure 2), which results in the oxidative damage to intracellular macromolecules and consequently leads to cell death. The mode of PDT-induced cell death is usually a mixture of apoptosis, necrosis and autophagy, with the dominance of a particular process depending on the PS (mainly its subcellular localization) as well as light fluence. It is generally agreed that apart from the direct cellular cytotoxicity, two other important factors contribute to the overall PDT effect: the vascular shutdown and local inflammatory reaction [1,2,3,4]. One of the major advantages of PDT over other anticancer treatment modalities is its high degree of selectivity. This is accomplished via the combination of two inactive components, visible light and a photosensitizing drug, which applied together in the presence of oxygen lead to generation of cytotoxic intermediates that effectively kill tumor cells [5].

As the PS alone is non-toxic and ineffective, to some extent it can be considered as a prodrug. However, additional selectivity of PDT may be achieved by the administration of a PS precursor. The only clinically approved example of such a compound is δ-aminolevulinic acid (ALA), a precursor of the natural photosensitizer phrotoporphyrin IX (PpIX). In contrast to exogenously administered PSs such as Photofrin, the photodynamically inactive, non-selective and non-toxic compound, ALA, is intracellularly metabolized to the photodynamically active PpIX. Subsequent illumination of the tumor site with red light activates PpIX, triggers the oxidative damage and induces cytotoxicity [6]. 

ALA is a naturally occurring compound, the early intermediate in the heme biosynthesis pathway. For therapeutic purposes ALA is administered topically or systemically and penetrates non-selectively into all cells, where it is metabolized to an active sensitizer PpIX. The bioactivation of ALA utilizes the enzyme machinery of the heme biosynthesis pathway. Although nearly all human cell types express the enzymes involved in the heme synthesis, a distinct activity of the enzymes in tumor as compared with normal cells leads to a higher PpIX accumulation within transformed cells [7].

For the last two decades a substantial amount of research has been focused on the elucidation of the mechanism of ALA-PDT and the improvement of its therapeutic activity. ALA is a polar molecule and in physiological pH occurs mainly as a charged zwitterion, which accounts for its low lipid solubility and reduced bioavailability. Further modifications of ALA aimed at improving its cellular permeability, increased stability in physiologic pH, increased selectivity and limitation of side effects, are important challenges in order to extend the clinical use of ALA-PDT. Since the very first topical application of ALA in the treatment of basal cell carcinoma in 1990 [8], the clinical use of ALA-PDT is still growing. Nowadays, PDT with ALA and its esters is an approved treatment of several malignant and premalignant conditions such as actinic keratosis, basal cell carcinoma, Bowen’s disease, bladder cancer and others. This review presents the use of ALA and its derivatives as prodrugs in PDT and summarizes the preclinical and clinical results of the treatment. 

## 2. Metabolism of ALA

### 2.1. Heme Biosynthesis

**Figure 3:**
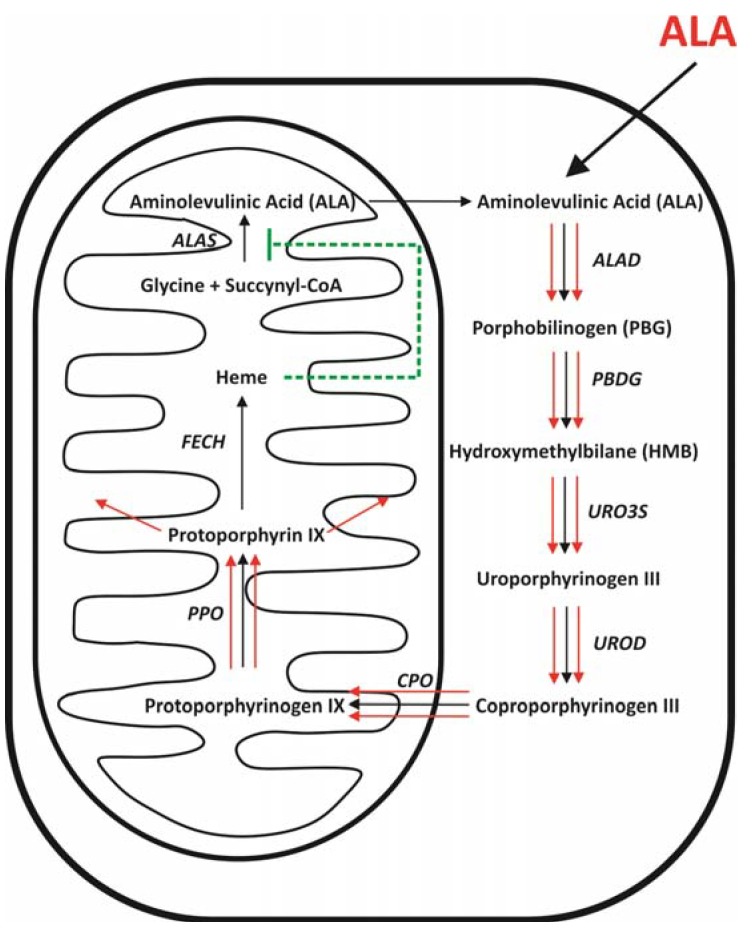
Heme biosynthesis. The graphic presents the most important steps in the heme biosynthetic pathway.

The synthesis of ALA is the first and rate-limiting step in the biosynthesis of heme [9,10,11]. ALA is normally synthesized in mitochondria in the condensation reaction between glycine and succinyl-CoA (Figure 3). The reaction is catalyzed by ALA synthase (ALAS) and requires pyridoxal-5-phosphate (PLP) as a cofactor. In mammals, two isoforms of ALA synthase have been identified: ALAS1, which is a housekeeping enzyme and ALAS2, which is expressed only in erythroid precursors [12]. 

After being synthesized ALA reaches cytosol, where it undergoes a condensation reaction. The reaction occurs between two ALA molecules with the aid of zinc-dependent enzyme – aminolevulinate dehydratase (ALAD) – and leads to the formation of porphobilinogen (PBG). ALAD, also known as porphobilinogen synthase (PBGS) comprises four homodimers, each of them having one active site [13]. Two molecules of ALA bind non-symmetrically to the active site, finally leading to the synthesis of PBG [14]. 

The next step in heme biosynthesis involves combining four molecules of PBG to form an unstable tetrapyrolle - hydroxymethylbilane (HMB). The reaction is catalyzed by porphobilinogen deaminase (PBDG), an enzyme containing dipyrromethane in its active site. Dipyrromethane is a co-factor covalently bound to the enzyme and consists of two PBG molecules. Four additional molecules of PBG attach to dipyrromethane leading to the formation of hexapyrolle. Afterwards, in the hydrolytic reaction, cleavage of the distal tetrapyrolle occurs, resulting in the release of HMB [15]. Hydroxymethylbilane can then enter two pathways. The first one uses uroporphyrinogen III synthase (URO3S) to close the HMB macrocycle leading to conversion of tetrapyrolle to uroporphyrinogen III. Alternatively, HMB can undergo spontaneous cyclization, which leads to the formation of uroporphyrinogen I [16].

Uroporphyrinogen decarboxylase (UROD) catalyzes decarboxylation of all four acetate side chains of uroporphyrinogen III to methyl groups [17]. The product of this reaction – coproporphyrinogen III is transported to the intermembrane space of mitochondria probably by peripheral-type benzodiazepine receptors (PBR) [18,19]. Coproporphyrinogen oxidase (CPO) then catalyzes the conversion of coporoporphyrinogen III to protoporporphyrinogen IX with the release of H_2_O_2_ and CO_2_. The reaction provides vinyl groups by oxidative decarboxylation of propionate groups on pyrolle rings A and B [20]. There are two forms of CPO: oxygen-dependent found in aerobic organisms, and oxygen-independent, in anaerobic and facultative bacteria [21]. In mammals, oxygen-dependent CPO is originally synthesized in cytosol with unusually long *N*-terminal sequence targeting it to the mitochondria [22]. Finally, CPO is situated in the mitochondrial intermembrane space with a fraction loosely bound to the inner membrane [23]. 

The next intermediate in heme biosynthesis, protoporphyrin IX (PpIX), is synthesized in the mitochondria and requires FAD-containing protoporphyrinogen oxidase (PPO) [24,25]. PPO catalyzes conversion of protoporphyrinogen IX to PpIX in the six-electron oxidation. The reaction requires oxygen as a terminal electron acceptor and leads to removal of six hydrogens from protoporphyrinogen IX [7]. Ferrochelatase (FECH), another rate-limiting enzyme, is responsible for insertion of Fe^2+^ into PpIX. The reaction occurs on the inner surface of the inner mitochondrial membrane [26]. This stage leads to the formation of the final product – heme – and completes the heme biosynthetic pathway. 

### 2.2. Heme Degradation

The enzyme responsible for heme degradation is heme oxygenase (HO). HO exists in two izoforms, significantly different in their regulatory mechanism: HO-1 is an inducible form whereas HO-2 is expressed constitutively [27,28]. They both catalyze the same reaction, which leads to the production of biliverdin, carbon monoxide (CO) and iron [29,30]. The reaction involves formation of HO-heme complex and reduction of ferric heme-iron to Fe^2+^ by NADPH:cytochrome p-450 reductase [31]. Afterwards, three oxygenation cycles occur leading to the production of ferribiliverdin IXa complex [32,33]. The iron of the complex undergoes reduction, which results in the release of free iron and biliverdin [31] Then, biliverdin can be further reduced to bilirubin by NADPH-dependent biliverdin reductase [34].

### 2.3. Effect of Exogenous ALA Administration

PpIX is a strong photosensitizer, which assembles in mitochondria of tumor cells leading to their damage after light exposure. Although all enzymes involved in the heme biosynthetic pathway are necessary, only two of them: ALAS1 and ferrochelatase are considered to be rate-limiting. Normally, the activity of ALAS1, is regulated by heme through the negative feedback mechanism. Heme binds to the heme-regulatory motif (HRM) in mitochondrial targeting sequence of ALAS and therefore prevents transport of ALAS1 precursor to mitochondria [35]. Moreover, there is evidence that heme not only regulates ALAS1 mitochondrial import but also attenuates its transcription [36,37]. Normally, the feedback mechanism leads to the production of PpIX in such amounts that can be efficiently converted to heme by ferrochelatase. Exogenous administration of ALA bypasses the natural regulation that heme exerts on ALA synthesis, which leads to increased production of PpIX [7]. The efficacy of ferrochelatase is then too low to convert excessively produced PpIX to heme, which results in the accumulation of PpIX within cells. About 4-6 hours after administration of ALA, when PpIX is already synthesized, target cells are exposed to light, which leads to excitation of the photosensitizer and formation of ^1^O_2_ that exerts cytotoxic effects.

### 2.4. Selectivity of PpIX Accumulation in Tumor Tissue in Response to ALA Administration

PpIX has been found to preferentially accumulate in tumor as compared with normal cells. This phenomenon can be explained by differences in heme biosynthetic pathway between non-malignant and malignant cells. It has been shown that decreased activity of ferrochelatase [38,39,40,41] and limited availability of iron [42] in tumor cells contribute to increased PpIX accumulation. Enhanced activity of enzymes leading to production of PpIX, such as ALAD [43], UROD [43], or PBGD [38,43,44] has also been observed in tumor cells.

### 2.5. Modifications of Heme Biosynthetic Pathway and Its Influence on ALA-PDT

Many studies have been performed in order to better understand the significance of individual parts of heme biosynthesis for ALA-PDT. The role of ALAD has been demonstrated by Feuerstein *et al*. who showed that silencing this enzyme with shRNA decreased synthesis of protoporphyrin IX in K562 erythroleukemic cells and resulted in impaired PDT outcome [45]. Similar effects have been presented for inhibition of PBGD with Pb^2+^ [46]. Furthermore, it has been revealed that pretreatment of LNCaP tumor cells with methotrexate significantly improved ALA-PDT efficacy. This effect was related to increased synthesis of coproporphyrinogen oxidase, which stimulated the accumulation of PpIX [47]. Moreover, studies in DBA/2 mice pretreated with PPO inhibitor revealed increased accumulation of cytosolic PpIX which potentiated the effect of ALA-PDT [48].

A significant amount of research has focused on the influence of ferrochelatase activity on the efficacy of ALA-PDT. Improved PDT outcome has been shown both after targeted knockdown of ferrochelatase by siRNA [49] and after pretreatment of tumor cells with ferrochelatase inhibitors [50]. Enhancement of PDT effects has been also observed after removal of iron with different iron-chelating substances such as desferrioxamine [51] or CP94 [52]. 

All these data suggest that efficacy of ALA-PDT is mainly regulated by amount of protoporphyrin IX. Therefore, attempts directed either towards its increased production or slower conversion to heme seem to be rational approaches to improve the outcome of the therapy. 

## 3. ALA Pharmacokinetics

Although a detailed summary of ALA pharmacokinetics is beyond the scope of this review, several issues are briefly outlined here. ALA is usually administrated intravenously (i.v.) or topically. For the treatment of bladder cancer ALA is administered intravesically. Moreover, in contrast to other PS, oral ALA administration is also feasible. Although this route is preferred by patients, the bioavailability of oral ALA is generally lower then after intravenous administration owing to presystemic drug elimination [53]. Large biosynthetic PpIX capacity of gastrointestinal mucosal cells and hepatic first pass metabolism are two major factors responsible for the reduction of ALA bioavailability. Indeed, Dalton *et al.* observed that only 60% of orally administered ALA is absorbed [54]. Regardless of poor oral ALA availability, the tissue PpIX concentrations were comparable after i.v. or oral administration [54]. On the other hand, preferential mucosal accumulation of PpIX might be favorable in the treatment of tumors of the gastrointestinal tract. Also, limited PpIX accumulation in the underlying stroma may reduce damage of deeper layers and risk of perforation or stenosis [55]. In terms of topical ALA administration it should be emphasized that due to the fact that *stratum corneum* is the most important barrier for ALA skin penetration, the PpIX fluorescence is observed only at a depth of 0.3 to 0.6 mm [56,57,58]. Therefore, a number of studies have focused on ALA derivatization to enhance its permeability through the lipid networks in the *stratum corneum* [59]. Moreover, since hyperkeratosis is an important negative factor in ALA uptake pretreatment with keratolytics, curettage/debulking, tape stripping, microdermabrasion or laser ablation can be used to improve ALA penetration [60]. 

In patients with bladder cancer, the intravesical ALA application results in pharmacokinetic advantages in comparison with oral administration. The bladder concentration of ALA is approximately 20,000-fold higher in comparison to the systemic circulation. Moreover, only 1% of the intravesical dose is absorbed from the bladder by the systemic circulation. Therefore, after intravesical ALA administration no systemic phototoxicity should be observed [54]. 

Identification of additional factors that affect ALA pharmacokinetics is necessary to design most effective ALA-PDT. Changes of numerous parameters of tumor cells as well as their surrounding microenvironment may alter PpIX production in malignant lesion. For example, the influence of tissue temperature on PpIX accumulation was observed in several studies [61,62]. Other parameters that affect PpIX accumulation include pH [63,64,65], oxygen availability [63,66], illumination conditions, or thickness of *striatum corneum* [67].

## 4. Modifications of ALA

Apart from a great potential and numerous advantages of ALA as a PS precursor, a number of limitations have also been revealed. ALA has a hydrophilic character and does not penetrate efficiently through the skin nor cell membranes. In consequence the production of PpIX after topical ALA administration is restricted to a 2–3 mm surface of skin, which might be insufficient to elicit satisfactory photosensitization [6]. Moreover, hyperkeratotic lesions drastically diminish ALA penetration through the skin. Since the hydrophilic nature of ALA limits its penetration ability restricting the use of ALA-PDT extensive studies were performed in order to facilitate its topical delivery. At least 77 various ALA modification and different carriers have been reported [68]. 

The most successful ALA derivatives are its esters: methyl ester (methyl aminolevulinate, MAL) and hexyl ester (hexyl aminolevulinate, HAL). Elongation of a carbon chain attached to ALA results in increased lipophilicity and in consequence higher membrane and skin permeability. The advantage of ALA derivatives over ALA can be mainly ascribed to: (i) the rate at which these compounds reach the target site, (ii) the rate at which they reach the intracellular space and (iii) the rate of their enzymatic conversion into photoactive compounds. Basically, these modifications are favorable and confer improved skin penetration. Nevertheless, extensively lipophilic agents tend to accumulate in *stratum corneum* which results in a drop in their biological activity*.* Different PpIX production profiles as a function of drug concentration have been observed for ALA and its esters. ALA and MAL profiles are identical, but HAL profile is completely different reaching plateau value at lower concentration [69]. Research by Lee *et al.* clearly shows that ALA butenyl, pentenyl and hexenyl esters induce higher production of PpIX than ALA or MAL [70]. In order to obtain comparable amount of PpIX with ALA-induced porphyrin accumulation, approximately 100 times less concentration of ALA heptyl ester is required [71]. On the other hand, undecanoyl-ALA ester, which reveals favorable diffusing properties, results in lower PpIX production than ALA itself. Even with the liposomal formulation it reaches only standard ALA-mediated PpIX level [72]. The advantage of ALA esters in comparison to ALA-inducedPpIX formation is not a general rule, some researchers report that it depends on esterase activity which varies within the tissues or cell lines used in the studies [73,74]. 

Administration of liposomes entrapping the ALA prodrug into tumor-bearing mice results in increment porphyrin biosynthesis as well as higher tumor to normal cells porphyrin ratio [75]. *In vitro* experiments demonstrated lipid sponge forms (thermodynamically stable, amphiphilic liquid) as a potential carrier for transdermal drug delivery [76]. It was also suggested that addition of dimethyl sulfoxide (DMSO) and ethylenediaminetetraacetic acid (EDTA) to ALA may enhance its delivery to hairless mice skin for topical PDT [77]. Conjugating 5-aminolevulinic acid to nanoparticles, including biocompatible gold (Au) and chitosan, was one of the solutions suggested to improve ALA delivery to the tumor [78,79]. Interestingly, Moan's group proposed topical bioadhesive patch systems that enhance selectivity of PpIX accumulation in tumor-bearing mice model [80]. 

## 5. Comparison of ALA with Porphyrin-based Photosensitizers

An ideal photosensitizer should be a well-defined chemical compound characterized by a strong selective phototoxic effect and an ability to generate active forms of oxygen. Moreover, it should show high absorption coefficients in the red part of the electromagnetic spectrum (600–900 nm) allowing the light to penetrate the tissue deeply. Its rapid clearance and favorable ADME (absorption, distribution, metabolism, excretion) parameters are also of utmost importance. The demand for such a compound is a challenge that leads to a synthesis of numerous photosensitizers. They differ from one another in terms of chemical structure, which often determines their pharmacokinetics, intracellular localization [81] and cytotoxic effect [82]. Generally, the currently used and investigated photosensitizing agents can be classified in two groups: porphyrin derivatives and non-porphyrin-based photosensitizers. The porphyrin family can be divided into three generations: the first one refers to photosensitizers developed between 1970 and 1980 and includes hematoporphyrin derivative (HpD) and Photofrin^®^. The second generation includes porphyrin derivatives that were intended to overcome the limitations of the former and the third generation encompasses photosensitizing agents conjugated with antibodies and other biological targeting molecules. So far only five photosensitizers have been approved for clinical PDT – porfimer sodium (Photofrin^®^), 5-aminolevulinic acid (ALA, Levulan^®^), and its methyl ester MAL (Metvix^®^), temoporfin (Foscan^®^), verteporfin (Visudyne^®^) and talaporfin (Laserphyrin^®^; the latter only in Japan). ALA is the only photosensitizer registered for topical use, particularly useful for the local treatment of superficial skin lesions. ALA-PDT has demonstrated high efficacy, minimal side effects and excellent cosmetic effects in a wide range of benign and malignant conditions.

ALA has several advantages over other photosensitizers, namely rapid metabolism and high selectivity for malignant lesions. Prompt systemic clearance of ALA-induced PpIX within 24 h eliminates prolonged photosensitivity and allows treatment to be repeated at regular intervals (as frequently as every 48 h) without cumulative effects and risk of damage to normal tissue. Nevertheless, significant disadvantages of ALA-PDT include pain associated with treatment [83], limited depth of tumor penetration [84,85], as well as individual variations among patients that affect ALA absorption and pharmacokinetics that influence effective ALA concentrations in the treated area [86]. Moreover, ALA-PDT appeared to be less efficient in destroying cutaneous lesions when compared with Photofrin-PDT [86]. A general comparison of ALA and different porphyrin-based photosensitizers, taking into account their physical and chemical properties as well as molecular mechanisms associated with the influence on tumor cells is provided in Table 1. 

**Table 1:** Properties of selected photosensitizers.

**Chemical classification**	Photosensitizer/trade name/company	Clinical approval/ clinical trials	Cellular localization	Advantages	Disadvantages	
**Hematoporphyrin**	Porfimer sodium - combination of monomers, dimers and oligomers of hematoporphyrin derivative (around 85% oligomeric material and mixture of more than 60 compounds)/ Photofrin /Axcan Pharma, QLT Pharmaceuticals	Approved	Plasma membrane Golgi apparatus [139,140]	Most commonly used photo-sensitizer, the longest clinical history and patient record, pain-free treatment	Complex composition, slow clearance rate, prolonged photosensitivity up to 3 months, low fluorescence quantum yield, low efficiency in ROS generation, limited penetration and efficacy in deep and bulky tumors	
**Protoporhyrin**	Pro drug (5-aminolevulinic acid - ALA) converted to photoactive protoporhyrin IX/ Levulan, Levulan Kerastick (for topical use)/ Dusa Pharmaceuticals)	Approved	Mitochondria, cell membranes, cytosol, cytosolic membranes [141]	Easy synthesis and formulation, minimal photosensitivity for no more than 24 hours (rapid clearance), excellent cosmetic results, especially eyelids, inexpensive, possibility of application without doctor’s supervision (oral and topical administration), can be administered at regular intervals (even every 48h), high selectivity due to metabolism of ALA in malignant cells and pilosebaceous units	Pain associated with treatment (need of local analgesia), need of prolonged contact period before illumination
	Aminolevulinic esters/ Metvixia/Galderma Benzvix, Hervix used for photodiagnosis [142] (PhotoCure AS)	Approved		Improved skin penetration - greater selectivity	Painful treatment
**Texaphyrins**	Motexafin lutetium/ Lutrin, Optrin, Antrin/ Pharmacyclics	Completed clinical trials phase I	Primarily in lysosomal compartment [143]	Deep tissue penetration	Severe pain during the phototherapy (need of local anesthesia)
**Porphycenes**	Various porphycene derivatives, modifiable isomers of porphyrin/ NDA	Phase II clinical trial of topical ATMPn (9-acetoxy-2,7,12,17-tetrakis(β-methoxyethyl)-porphycene)	Mitochondria LysosomesER plasma membrane [144]	Efficient ROS generation, possibility of various structural and chemical modifications that improve half-life and enhance therapeutic efficiency	Photobiological properties still poorly explored
**Purpurins**	Tin etyl etiopurpuryn rostaporfin/ SnET2, Photrex, Purlytin/ Miravant Medical Technologies	In clinical trials phase III	Mitochondria lysosomes	Excellent cosmetics effect, effective in treatment of locally advanced metastatic malignancies [145]	Post-treatment pain, long-lasting photo-sensitization up to 14 days [146], poor stability in water (need of formulation in lipid emulsions, which can lead to allergic reactions)
**Pheophorbides**	WST-09 (padoporfin, palladium bacteriopherophorbide /Tookad and WST-11(padeliporphin) /Stakel/ Steba Biotech	WST11- phase I and II, WST-09 – phase II	NDA	Little or no skin-associated sensitivity, greater tissue penetration [84], the possibility of repeated treatments	Narrow time window available for light delivery (important in clinical settings)
**Chlorins**	Talaporfin sodium, mono-L-aspartyl chlorine 6/ NPe6, MACE, LS11, Laserphyrin, Photolon, Aptocine/ Light Sciences Oncology	Approved	Lysosomes	Excellent singlet oxygen yield, used in Litx therapy (Light Infusion Technology), where talaporfin is illuminated for prolonged time (1–3 h) locally with light-emitting diodes (LEDs) implanted in the tumor, minimal skin photo-sensitivity	In clinically needed high doses little selectivity to tumor tissue occurred [147]	
	Temoporfin, meta-tetrahydroxyphenylchlorine, mTHPC/ Foscan/ Biolitec Pharma	Approved	ER mitochondria	High singlet oxygen yield (low drug dose and low light dose -20J/cm2 are required to generate photodynamic reaction), low activation energy and short time treatment, long half-life in triplet state	Accumulation in the skin, requires strict protection of the eyes and skin from sunlight for up to 6 weeks, long drug-illumination interval, requires very precise illumination (reflected light can produce photodynamic reaction) and accurate dosimetry	
	HPPH 2-(1-hexyloxyethyl)- 2-devinyl pyropheo-phorbide/ Photochlor/ Roswell Park Cancer Institute	In naturally occurring veterinary tumors (cats and dogs), clinical trials phase I and II	Mitochondria	Minimal sunlight photosensitivity, relatively easy to synthesize	Phototoxicity not determined in higher doses	
**Phthalocyanines **	Aluminium (III) phthalo-cyanine tetrasulphate, AlPcS4/Photosens (mixture of sulfonated aluminium phtalocyanines)/ developed in Russia, General Physics Institute	In naturally occurring veterinary tumors, several clinical trials in Russia	Mitochondria	High singlet oxygen yield with long-lived triplet states due to presence of aluminum atom, due to enhance fluorescence can be used for diagnostic purpose, minimal photosensitivity	Problems with purification, typically final product is a mixture of mono- di- tri- and tetrasulphonated derivatives, in water aggregate at relatively low concentrations which results in loss of photo-chemical activity	
	Silicon phthalocyanine 4/ Pc4/ Case Western Reserve University	Ongoing clinical trials phase I	Mitochondria ER	High singlet oxygen yield with long-lived triplet states due to presence of silicon atom, good efficacy in both preclinical and clinical studies, due to enhance fluorescence can be used for diagnostic purposes	Heterogeneous distribution within and between lesions detected by noninvasive spectroscopy [148]	
**Benzoporphyrins**	Verteprofin/Visudyne /Novartis	Approved	Mitochondria and ER	Deep tissue penetration, minimal photo-sensitization up to 48 h, effectiveness in neovascular lesions, successful in cutaneous lesions	Painful administration [149]	

Abbreviations used: ER – endoplasmatic reticulum, NDA – no data available, ROS – reactive oxygen species

## 6. Preclinical Studies with ALA

The first report on excessive protoporphyrin accumulation and high photosensitivity after exogenous administration of ALA in humans was presented by Berlin *et al.* in 1956 [87]. Several years later detailed *in vitro* studies of endogenous photosensitization in Friend erythroleukemic cells were done by Mailik and Djaldetti [88]. In 1987 ALA was used for the first time in photodynamic therapy as a porphyrin precursor for selective elimination of erythroleukaemic cells [89]. In the same year Peng *et al.* presented the results of an *in vivo* study showing porphyrin accumulation and fluorescence in tumors and other tissues in mice 24h post intraperitoneal injection of ALA [90]. In 1990 Kennedy and Pottier reported the earliest clinical trial with ALA-PDT for superficial basal cell carcinomas [91].

Subsequent *in vitro* and *in vivo* studies were performed to find the best parameters for drug administration and light exposure [62]. In 1992 a selective accumulation of endogenously produced porphyrins in a liver metastasis model in Wag/Rij rats was reported. Porphyrin concentration was increasing in tumors with prolonged duration of ALA oral administration (in drinking water), whereas no augmentation was found in healthy livers [92]. *In vitro* measurement of the fluorescence distribution and biological effect of induced PpIX in normal rat colon cells and colon carcinoma showed good correlation between the fluorescence distribution and the biological effect of ALA-induced photosensitization on exposure to red light. Detected significant differences in PpIX concentration in tumors as compared with normal mucosa and normal muscle suggested more selective necrosis in tumors [93]. Similar study was performed in order to characterize ALA fluorescence in numerous tumor cell lines including oral squamous cell carcinoma [94], human urothelial carcinomas [95], human hepatoblastoma and neuroblastoma cells [96]. 

*In vivo* efficiency of photodynamic diagnosis and therapy is determined by various parameters such as PpIX concentration and distribution, fluorescence rate, treatment wavelength, ﬂuence and oxygen availability. Several investigations were conducted in order to optimize these parameters in a rat Barrett’s esophagus model [97,98,99]. The kinetics and spectra of ALA-induced fluorescence were determined in tumor and surrounding tissue of amelanotic melanoma in hamsters [100], various mammary carcinoma bearing mice [19,20], superficial bladder carcinoma in rats [101], human hepatocellular carcinoma cell [102], human nasopharyngeal carcinoma and human rectal cancer in nude mice [103,104]. Prolonged animal survival followed by photodynamic therapy using ALA was reported by several groups in various experimental models. One of the first effective *in vivo* PDT treatments was shown in 1994 by Regula *et al.* in a pancreatic cancer model in Syrian golden hamster. Further investigation revealed similar efficiency after ALA-PDT in orthotopic urothelial carcinoma in Fisher rats, rat high-grade gliomas, human cervical cancer and human gastric cancer in nude mice [105,106] .

Even though ALA-PDT was intensively studied for last decades, still little is known about the mechanism of its cytotoxicity. For ALA-PDT Grebenova *et al*. proposed two different, mitochondrial and endoplasmic reticulum stress-induced, cell death pathways [107]. It was reported that ALA-PDT induces apoptosis in human glioma cells through the mitochondrial cell death pathway. Depolarization of the mitochondrial membrane potential and the release of mitochondrial cytochrome c and caspase activation upon ALA-PDT were confirmed [107]. In HL60 leukemia cells ALA-PDT initiated several signaling processes that led to rapid induction of apoptosis, followed by slow necrosis [108]. It was revealed that after ALA-PDT, K562 cells displayed apoptotic features such as mitochondrial membrane disintegration and mitochondrial cytochrome c release. However, there was no caspase-3 activation or DNA fragmentation leading to apoptosis process termination. Finally, cells died by necrosis as a consequence of plasma membrane damage [109]. These divergent observations seem to result from the fact that ALA-PDT leads to apoptosis when PpIX accumulates mainly in mitochondria and to necrosis when it diffuses to other cellular compartments such as cytoplasm [50]. Interestingly, Ji *et al.* recently reported that besides apoptosis and necrosis ALA-PDT might mediate autophagic cell death. Further observations indicate that the AMPK pathway plays an important role in ALA-PDT induced autophagy in PC12 and CL1-0 cells [110]. 

## 7. Clinical Indications for ALA and Its Esters

Noninvasive therapy with ALA seems to be an attractive treatment modality for patients with comorbidities and high risk of the surgery. Moreover, due to good cosmetic effects of this therapy, ALA and its esters are commonly used in dermatology. Currently, most clinical studies on ALA-PDT are focused on the treatment of nonmelanotic skin cancers and cutaneous T-cell lymphoma. The clinical efficiency for superficial basal cell carcinoma estimates from 50% to 100% complete responses and the effects of the therapy improve due to the implementation of fractional PDT sessions [111,112,113]. Unfortunately, 60 months after the treatment the recurrence rate reaches as much as 43% [114]. On the other hand, in the treatment of cutaneous T cell lymphoma Coors and colleagues were able to achieve 100% response with no recurrence after 18 months [115]. The encouraging results are also obtained in the treatment of invasive squamous cell carcinoma indicating that compared with cryotherapy, ALA-PDT was more effective and better tolerated by patients [116]. Additionally, in the clinical study of multiple actinic keratoses of the face and scalp, topical ALA-PDT was shown to be effective and safe. 

Complete response at 12 week was observed in above 80% of patients, while the negative effects of therapy, like skin sensitivity and light-burning, decreased or resolved 24 hours after the illumination [117]. In phase IV clinical trials after 12 months of therapy the recurrence rate was 19% [118]. Moreover, ALA-PDT was shown to be an effective treatment for head and neck cancer, especially for superficial lesions [119]*.* Few other reports revealed complete or partial response to ALA-PDT in patients with oral cavity, oral verrucous hyperplasia, larynx and hypopharynx carcinoma [120,121,122]. ALA is also commonly used in the treatment of precancerous lesions of gastro-intestinal tract. The therapies of high-grade dysplasia and metaplasia of Barrett’s esophagus rely on preferential uptake of ALA, which is 3.5 times greater in mucosa then in muscles [123]. ALA-PDT was shown to be more effective in eradicating high-grade dysplasia than in treatment of early esophagus cancer [124,125].

MAL was approved in the treatment of actinic keratosis and nonmelanoma skin cancers (basal cell carcinoma) in Australia, New Zealand and some European countries. MAL, administered in a form of a cream, 3 hours after application under occlusion is activated with red light usually in a total dose of 75 J/cm^2^. MAL is known to be more selective then ALA. Moreover, the therapy is comparably or less painful and causes less systemic effects after local treatment [126,127,128]. Compared with cryotherapy for actinic keratosis MAL-PDT was found to have a better or similar effectiveness (complete response rate 68%–75% after single treatment) [129,130]. However, the outstanding cosmetic effects after MAL-PDT bring more satisfying results for patients [131].

The results obtained with MAL-PDT particularly depend on the level of pigmentation and the size of the lesion. Therefore in the treatment of basal cell carcinoma (BCC) much better response is reached for superficial BCC than nodular BCC [132,133]. This is the reason why the optimization of PDT procedure requires selection of the lesions. However, in the treatment of large lesions or more aggressive lesions with H-zone in patients with high risk of surgery, Viniciullo *et al.* received a 90% of complete response after 3 months, which had decreased to 78% following next 18 months [134]. The satisfying results are also obtained in the treatment of cutaneous T cell lymphoma. However, due to the limitation of MAL in penetration into the deeper layers of tissues, this disease appears to be more resistant to MAL-PDT [135]. Currently another ester of ALA, the hexyl ester (HLA) is in a clinical study of the diagnosis and treatment of bladder cancer. Compared to ALA, the HLA was shown to have similar fluorescence intensity, depth of penetration, and distribution in basal cell carcinoma. However, the important advantage of HLA is a possibility to administer this pro-drug in a lower concentration than those used for ALA [136].

## 8. Limitations of ALA-PDT

The most important drawbacks of PDT are associated with light hypersensitivity, inefficiency in the management of large or metastatic tumors and pain during treatment. Limited light penetration through tissues makes this treatment ineffective in the treatment of bulky or disseminated tumors. Additionally, topical, and to some extent also systemically administered ALA has rather limited ability to penetrate tissues further restricting the efficacy of treatment. Although ALA-PDT is generally well tolerated, some patients complain of stinging, burning, pricking, smarting and itching pain during PDT and several hours after irradiation. Pain can be relieved with topical anesthetics, but only those that have neutral or acidic pH as ALA becomes unstable in alkaline environment [83]. A disagreeable pain or a history of previous experience of long-lasting pain may be managed with cyclooxygenase antagonists [137], especially that COX-2 inhibitors seem to potentiate antitumor effects of PDT [138]. Occasionally patients may develop postinflammatory changes in pigmentation, that are usually transient. Other rare adverse effects associated with ALA administration include nausea, fatigue, paraesthesia and headache. 

## 9. Summary

Although ALA-PDT is considered to be a highly promising antitumor strategy it still necessitates several areas of ongoing pre-clinical and clinical investigations. Its advantages over traditional antitumor treatments such as surgery, chemotherapy or radiotherapy, include lack of intrinsic resistance mechanisms to ^1^O_2_-induced cytotoxicity, reduced long-term morbidity and the possibility of repeated treatment. Finally, many conventional antitumor treatments carry the risk of inducing immunosuppression, which is not associated with the use of PDT. Detailed studies on the molecular mechanisms of cytotoxicity are necessary to better understand therapeutic outcomes that could be translated into more effective therapeutic regimens. Altogether, ALA-PDT seems to be the most successful pro-drug treatment in clinical oncology.
